# Editorial Correspondence

**Published:** 1871-10

**Authors:** W. F. Westmoreland

**Affiliations:** New York


### EDITORIAL CORRESPONDENCE.

New York, September 30th, 1871.

*Dear Journal:*—Ten days ago, I made a hurried visit to Philadelphia and Baltimore, and in both cities met a number of old friends, and made many pleasant acquaintances.

My first visit, as is usual with me upon my arrival in Philadelphia, was to the halls of my Alma Mater, the Jefferson Medical College, where I found a very respectable class in attendance upon the preliminary course of lectures in that institution. Twenty years have made but little change in the old building, its halls appearing as familiar as on the day I received my degree; but alas! death has made sad havoc in that noble corps of teachers who, by their eloquence and learning, so captivated the five or six hundred students that crowded its halls at that time. The voices of Mütter, Meigs, Mitchell, Bache, Dunglinson and Houston are stilled in death; one after another they have passed away, and their places are filled by worthy and competent successors. There alone remains, of the Faculty of 1849–'50, that veteran teacher, Prof. Joseph Pancoast.

I had a long and pleasant interview with that great surgeon and accomplished teacher, Prof. S. D. Gross. Time has made but little impression on him, as he looks as well as three years ago. He is re-writing his work on Surgery, which is now passing through the press, and will appear some time next year.

I very much regretted to find Professor Pancoast absent from the city, but was consoled by a visit to that indefatigable laborer in his specialty, Dr. J. Solis Cohen. I spent several hours at his rooms, and was fully compensated for my visit. Dr. Cohen has, for many years, given almost his entire time to the diseases of the nasal cavities, throat and ear, and may now be regarded as the first authority in Philadelphia upon this class of disease. He is now Lecturer on Laryngoscopy and Diseases of the Throat and Chest in Jefferson Medical College, and has been appointed Mütter Lecturer in the College of Physicians of Philadelphia, and will deliver a course before that body the coming winter on "Surgical Diseases of the Air-Passages." He has also in press a work on the "Diseases of the Throat and Nasal Cavities, and their Modern Treatment," which will be issued in a few months from the publishing house of William Wood & Co., New York.

Notwithstanding his labors in the direction above indicated, he has found time to give some attention to electro-therapeutics, and more particularly to electrolysis. He has recently translated from the French M. Legros and Onimus' little work, "Observations on the Effects of the Electric Currents upon the Living Tissues and upon Nutrition," and by other contributions, added to the literature of the subject. His results with the galvanic current on tumors are encouraging, particularly in goitre, a class of cases in which he has had a better opportunity ot testing its powers than in any other.

I very much regretted that my stay in Philadelphia was so short—twenty-four hours—but previous engagements were imperious. I hope, however, to again visit the Quaker City before I return to Atlanta.

I found Baltimore crowded with strangers, attracted by the Masonic festivities, the Commercial Convention, and the Episcopal Convention of Bishops to assemble in a few days. Atlanta was well represented, both in point of talent and beauty.

The Medical Schools here—the University of Maryland and the Washington University—have not yet commenced their regular winter labors; both schools, however, are giving a preliminary course. From all that I could learn, there will be at least the average number of students in Baltimore the preasent season.

I had the pleasure of meeting quite a number of medical men in this city—among others, Dr. Nathan R. Smith, late Professor of Surgery in the University of Maryland. For many years, I have regarded Dr. Smith as one of the soundest medical philosophers on this continent. His suggestions have ever been rational and practical, evidently the result of mature deliberation. The splint that bears his name, "Smith's Anterior Splint," has done as much to alleviate suffering and preserve the normal functions of the limb in cases where applicable and properly applied, as any suggestion in bone surgery during the present century. Although extensively used in certain localities in this country, and well understood and extensively adopted in Europe, still there are localities and even extensive hospitals—Bellevue Hospital, for example—in our own country, where I have never seen it used, and by inquiry, I learn it is very rarely spoken of. During my present visit North, I have seen more than one case where I felt confident that "Smith's Anterior Splint," properly applied, would have saved the sufferer many days of agony, and greatly increased the chances of life and limb.

Although Dr. Smith is growing old, still he is as enthusiastic in all that pertains to his profession as when I first met him years ago. He has given up teaching, but is still actively engaged in his profession. May many long years of usefulness yet be spared him.

Dr. J. J. Chisolm, of Confederate memory, now Professor of the Diseases of the Eye and Ear in the University of Maryland, is a fixture in Baltimore. He is devoting himself exclusively to diseases of the eye and ear, and has already succeeded in establishing an enviable reputation and an extensive business in this department of surgery. He has recently established a private hospital for the treatment of the diseases of the eye and ear. It is gotten up in fine style, and its appointments are most excellent. This is a move in the right direction. The great difficulty in obtaining appropriate apartments and attendance at a hotel or boarding house, for the successful treatment of many of the diseases of the eye, is obvious to every medical man. In such an establishment as Dr. Chisolm's, where the patient is quartered in apartments best adapted to his or her peculiar condition, with attentive and trained nurses, the per centage of success in the treatment of many diseases is certainly greatly increased. Another important feature is, that the cost is much less to the patient than at hotels.

Dr. Edward Warren, late Professor of Surgery in the Washington University, has resigned his position in that institution. The institution has lost one of its best and most influential teachers. Dr. Warren, if I mistake not, is one of the founders of the Washington University, and has done as much or more than any one for the success of the enterprise. When in Baltimore three years ago, I had the pleasure of hearing him lecture, and was then forcibly impressed with his great excellence as a teacher. He is still actively engaged in his profession, and occupies an enviable position, not only in Baltimore, but throughout the country, as a surgeon and teacher.

I called upon Dr. Frank Donaldson, of the University of Maryland, and found him a much younger man than I expected to meet. I very much regret that a pressing call prevented my seeing as much of him as I desired. For several years past, he has given great attention to the diseases of the throat and nasal cavities, and is the authority upon that class of disease in Baltimore.

Upon a visit to the Washington University, I had the pleasure of meeting my friend, Dr. Chancellor, Professor of Anatomy and Dean of the Faculty. He speaks in very sanguine terms of the future of the institution.

Upon my return to New York, I was pleased to meet that distinguished surgeon from our own section, Dr. Warren Stone, of New Orleans, one whom all love to honor. He has just returned from Europe, and looks as young and vigorous as ten years ago. No one can come in contact, even for a short time, with Dr. Stone, but is impressed with his great intellect, his thorough investigation of all subjects connected with his profession, and the candor and sincerity with which he expresses his opinions. The profession has lost much by his indisposition to write, as all feel that he has done not only the profession, but himself, great injustice in not giving the results of his observations of the forty years of active professional life in the extensive field in which he has labored. It is not unusual to see, in our medical journals, suggestions as new and important, and accepted by the profession as original, when those in his immediate vicinity know that he has acted upon them for years.

Many here have attempted to impress Dr. Stone with the importance of giving to the profession, in some form, the results of his extended observations. With others, I suggested that in the event he could not find time to give to such pursuits, that he have a stenographer to follow him in his lectures at the college and hospital, and publish in the form of lectures. It is to be hoped that he will comply with the wish of his legion of admirers in every section of the country.

A few days ago, I had the unexpected pleasure of meeting my old friend, Dr. J. T. Gilmore, Professor of Surgery in the Mobile Medical College. The Doctor is here on a tour of recreation, that he may be better prepared to endure the arduous labors of the coming winter. I had not met him since we parted in Columbus, Mississippi, after the disastrous campaign of Hood's army into Tennessee. Six years have produced but little change in his appearance, and none in his devotion to his profession. He has an extensive field in Mobile, and I predict for him a brilliant future. In company with him, a few days ago, I visited Bellevue Hospital, to witness Dr. Gouley perform his favorite operation for stone in the bladder—lithotripsy. Dr. Gilmore and myself had frequently seen the great Civiale perform the same operation, and, in justice to Dr. Gouley, would say that we have never witnessed the operation performed with more facility, and with less apparent injury to the bladder and urethra. I had the pleasure of seeing the patient several times after the operation, and what is unusual, the first sitting reduced the stone and completed the cure.

Through the courtesy of Dr. J. Marion Sims, I, some days ago, witnessed the administration of nitrous oxide gas as an anæsthetic—not that there is anything new in the administration of this gas as an anæsthetic, as it has been frequently administered in surgical operations, but confined to such as only required a very few minutes to complete them. Dr. Sims is desirous of finding some agent that may be administered for a length of time—for instance, in such cases as ovariotomy—without the depression, nausea and vomiting that so constantly attend the administration of chloroform and ether. The operation was in a patient with great relaxation of the vagina and perinæum, with extreme prolapsus of the pelvic viscera, and consisted in partially closing the vaginal outlet and reducing the vaginal canal by denuding its posterior wall, and bringing the denuded surfaces together by means of the silver suture.

The operation within itself had no peculiar interest, but the long-continued use of this anæsthetic was new and interesting. The patient was kept fully under its influence for eighteen and a half minutes, and during the entire time, there was complete insensibility, without any unpleasant symptoms. The circulation was less affected than from the administration of chloroform or ether. There was slight nausea, but no vomiting. The patient inhaled fifty gallons of gas.

A few days later, he did me the honor to have me present when the nitrous oxide gas was administered in a case of hemorrhoids, in which he proposed to remove the hemorrhoidal tumors with the galvano-cautery. The nitrous oxide gas was administered, as in the above-mentioned case, to complete anæsthesia, and the patient kept under its influence for seventeen minutes. Like the other case, the circulation was very slightly, if at all, interfered with; but in this case, there was nausea and vomiting a very few minutes after the patient passed from under the induence of the anæsthetic. In this case, there was about forty gallons of the gas used.

In witnessing these two cases, I could not see why the gas could not be administered for any length of time that might be desired. The only difficulty that I noticed was the inconvenience of transporting the gas, and the formidable display, which would be rather trying to the nerves of some patients. In these two cases, the nitrous oxide was transported in an hundred-gallon rubber bag. Dr. Sims informed me that he had made arrangements to have the gas compressed, so that he could have compressed into a half-gallon jar, fifty gallons of the gas.

In consequence of an accident to the galvanic battery, I saw two modes—to me both new—of removing hemorrhoidal tumors. As above stated, Dr. Sims proposed to remove the tumors by the galvano-cautery. After expelling the tumors, the largest was grasped at its base with his modification of Henry Smith's clamp, and firmly compressed by means of the screw. The battery was now put in action, and the galvano-cautery blade attached to the electrode; in a few seconds the blade was at white heat, when the tumor was shaved off at from a twelfth to an eighth of an inch from the blades of the clamp. Just before the operation was completed, the battery or the electrodes became disarranged. Upon loosening the clamp, a small bleeding vessel was discovered; as the battery would no longer act, the little artery was ligatured. As the galvano cautery was no longer of use, the remaining tumor was removed by Dr. J. C. Nott, with his recti-linear ecraseur. As Dr. Nott has promised to prepare for the JOURNAL a description of his instrument, accompanied by a wood cut, and also the mode of its use, I will not attempt a description here.

I have had the pleasure of meeting Dr. Nott upon several occasions since I arrived in New York, and each time I was more favorably impressed with his fine intellect, his great learning, and his candor and honesty of purpose. Dr. Nott has only been in the city three or four years, and, without any fortuitous circumstances, has, by his own intrinsic worth, made for himself a position second to no medical man in the city.

In my last letter, I promised to further investigate the subject of electrolysis, and give my impressions as to what may be accomplished with the galvanic current. I have left nothing undone to inform myself upon the subject, but still the problem has not been solved to my satisfaction. I have frequently visited those most actively engaged in the study of electrolysis (I say study, as all are yet students)—have witnessed their experiments—have watched, with interest, the results of their operations—have listened, with great interest, to their explanations—and still there are many doubts in my mind in regard to the correctness of some of their theories, and what may be really accomplished by this agent. Of three facts there can be but little or no doubt:

1. That the galvanic current, when properly passed through a malignant tumor, sufficiently intense to induce chemical changes in the diseased mass, will promptly arrest that peculiar pain common to cancerous growths.

2. That the galvanic current is capable, when properly applied, of dissolving or otherwise causing a cancerous mass to disappear, without either inflammation or suppuration.

3. That when the current is passed through the original deposit, as in cancer of the mammary gland, the secondary glandular growths in the vicinity of the original, will decrease in size in proportion to the decrease in the size of the original deposit, and that with the cessation of pain and the decrease in the secondary growths, the general health will improve.

These are facts without deduction, which I have seen illustrated by several marked cases. In my next letter, I may be able to give you other facts.

Truly, W. F. WESTMORELAND.

